# Prevalence of Inflammatory Bowel Disease Among Medicare Fee-For-Service Beneficiaries — United States, 2001−2018

**DOI:** 10.15585/mmwr.mm7019a2

**Published:** 2021-05-14

**Authors:** Fang Xu, Susan A. Carlson, Yong Liu, Kurt J. Greenlund

**Affiliations:** 1 Division of Population Health, National Center for Chronic Disease Prevention and Health Promotion, CDC.

Inflammatory bowel disease (IBD), including Crohn’s disease and ulcerative colitis, is characterized by chronic inflammation of the gastrointestinal tract. The number of affected persons worldwide has increased from 3.7 million in 1990 to 6.8 million in 2017 (*1*). The disease is more prevalent among non-Hispanic White persons than it is among persons in other racial/ethnic groups (*2*). As the prevalence increases with age group (*2*), it is important to understand the disease epidemiology among the older population. CDC analyzed 2018 Medicare data among beneficiaries aged ≥67 years to examine differences by demographic characteristics for both diseases and to assess trends of prevalence from 2001 through 2018 both overall and by race and ethnicity. In 2018, 0.40% and 0.64% of 25.1 million Medicare fee-for-service beneficiaries aged ≥67 years had received a diagnosis of either Crohn’s disease or ulcerative colitis. Prevalence varied by age, sex, race and ethnicity, urban-rural residency, and state. During 2001−2018, the age-adjusted prevalence of both diseases increased (Crohn’s disease annual percentage change [APC] = 3.4%, ulcerative colitis APC = 2.8%). The increase was higher among non-Hispanic Black persons (Crohn’s disease APC = 5.0%, ulcerative colitis APC = 3.5%) than it was among non-Hispanic White, Hispanic, and Asian/Pacific Islander (A/PI) persons. Prevalence was consistently highest among non-Hispanic White persons for both diseases and lowest among A/PI persons for Crohn’s disease. The study findings of increasing prevalence in all racial/ethnic groups among older adults, especially the higher rate of increase among certain racial/ethnic minority groups, underscore the importance for promoting health equity, guiding efforts to tailor disease management strategies for different populations, and continuing to monitor the temporal trends of the disease.

CDC examined data for U.S. adults aged ≥67 years who were continuously enrolled throughout a calendar year during 2001−2018 in Medicare parts A and B* and who were not enrolled in a health maintenance organization plan. This included 25.1 million beneficiaries in 2018 and ranged during 2001−2018 from 23.7 million persons in 2009 to 25.6 million in 2005. Study participants were identified by using *International Classification of Diseases, Clinical Modification* diagnosis codes from the ninth (ICD-9-CM) and, after October 1, 2015, the tenth (ICD-10-CM) revisions. Crohn’s disease (ICD-9-CM: 555, ICD-10-CM: K50) and ulcerative colitis (ICD-9-CM: 556, ICD-10-CM: K51) were each identified by searching for any listed diagnosis code, including a 3-year look back, in Medicare part A data for at least one inpatient stay or in part B data for at least two claims with different dates. Beneficiaries with codes for both diseases were excluded (0.02% of all Medicare fee-for-service claims) to avoid possible disease misclassification. Variables included state of residence, age group (67–74, 75–84, and ≥85 years), sex, race/ethnicity (non-Hispanic White, non-Hispanic Black, Hispanic, non-Hispanic A/PI, and non-Hispanic American Indian/Alaska Native [AI/AN]), and urban-rural residency based on the National Center for Health Statistics 2013 classification scheme (large central metropolitan, large fringe metropolitan, medium metropolitan, small metropolitan, micropolitan, and noncore).^†^ Prevalence estimates and 95% confidence intervals (CIs) were calculated overall and by demographic subgroups for 2018. Group differences were determined by z-test with the significance level set at 0.05. Prevalence estimates were age-adjusted^§^ when presented by state and for all trend analyses. For 2001−2018, annual prevalence was estimated overall and by race/ethnicity. Trends were assessed by using linear regression models weighted by inversed standard errors. An interaction term for race/ethnicity and year was included to assess the differences in trends between racial/ethnic groups. Analyses were performed by using SAS Enterprise Guide (version 7.1; SAS Institute).

In 2018, 0.40% and 0.64% of Medicare fee-for-service beneficiaries aged ≥67 years had received a diagnosis of either Crohn’s disease or ulcerative colitis (Table). The prevalence of Crohn’s disease was higher among younger beneficiaries, highest among non-Hispanic White persons, and lowest among non-Hispanic A/PI persons. The prevalence of ulcerative colitis was highest among beneficiaries aged 75–84 years and among non-Hispanic White persons. For both diseases, prevalence estimates were higher among women than they were among men. Estimates for both diseases were highest among persons in large fringe metropolitan counties, second lowest among those in micropolitan counties, and lowest among those in noncore counties. Age-adjusted state-level prevalence estimates ranged from 0.17% (Hawaii) to 0.62% (Rhode Island) for Crohn’s disease and from 0.37% (Hawaii) to 0.91% (New Jersey) for ulcerative colitis. States with a higher prevalence of both diseases were generally concentrated in the Northeast (Figure 1).

**TABLE:** Prevalence of Crohn’s disease and ulcerative colitis among 25.1 million Medicare fee-for-service beneficiaries,* by age group, sex, race/ethnicity, and urban-rural residency — United States, 2018

Characteristic	Crohn’s disease,^†^ % (95% CI)	Ulcerative colitis,^§^ % (95% CI)
**No.**	**99,665**	**161,494**
**Crude rate**	0.40 (0.40−0.40)	0.64 (0.64−0.65)
**Age-adjusted** ^¶^	0.40 (0.40−0.40)	0.65 (0.64−0.65)
**Age group, yrs**		
67−74	0.42 (0.41−0.42)	0.60 (0.60−0.61)
75−84	0.41 (0.41−0.42)	0.70 (0.69−0.71)
≥85	0.32 (0.31−0.32)	0.65 (0.64−0.65)
**Sex**		
Male	0.36 (0.36−0.36)	0.61 (0.60−0.61)
Female	0.43 (0.43−0.43)	0.68 (0.67−0.68)
**Race/Ethnicity**		
White, non-Hispanic	0.43 (0.43−0.43)	0.69 (0.69−0.69)
Black, non-Hispanic	0.26 (0.25−0.27)	0.41 (0.40−0.42)
Hispanic	0.19 (0.18−0.20)	0.43 (0.42−0.44)
Asian/Pacific Islander, non-Hispanic	0.15 (0.14−0.15)	0.37 (0.36−0.38)
American Indian/Alaska Native, non-Hispanic	0.23 (0.20−0.26)	0.40 (0.36−0.43)
Urban-rural residency**		
Large central metropolitan	0.39 (0.38−0.39)	0.68 (0.67−0.69)
Large fringe metropolitan	0.46 (0.45−0.46)	0.76 (0.76−0.77)
Medium metropolitan	0.40 (0.40−0.41)	0.63 (0.62−0.63)
Small metropolitan	0.38 (0.37−0.39)	0.60 (0.59−0.61)
Micropolitan	0.36 (0.35−0.37)	0.54 (0.54−0.55)
Noncore	0.33 (0.32−0.34)	0.49 (0.48−0.50)

**Abbreviation**: CI = confidence interval.

* The estimated number of Medicare-eligible fee-for-service enrollees aged ≥67 years in 2017 was 25,069,000.

^†^ *International Classification of Diseases, Tenth Revision, Clinical Modification* diagnosis code K50.

^§^ *International Classification of Diseases, Tenth Revision, Clinical Modification* diagnosis code K51.

^¶^ Age-adjusted to the 2000 U.S. Census population aged ≥67 years based on three age groups (67–74, 75–84, and ≥85 years). https://data.census.gov/cedsci/table?t=Age%20and%20Sex&y=2000&d=DEC%20Summary%20File%201&tid=DECENNIALSF12000.PCT012&hidePreview=false

** Based on the 2013 National Center for Health Statistics urban-rural classification scheme for counties. https://www.cdc.gov/nchs/data/series/sr_02/sr02_166.pdf

**FIGURE 1:**
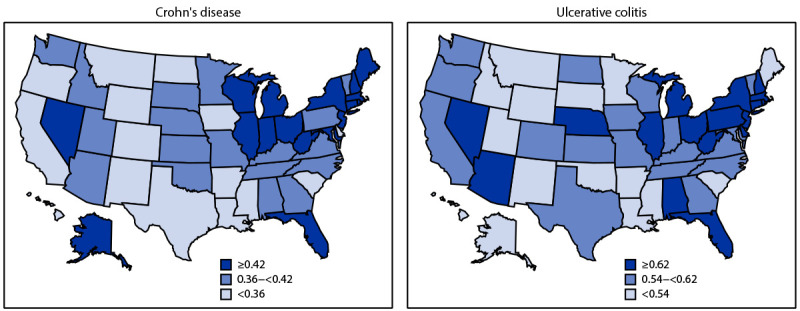
Age-adjusted prevalence*^,†^ of Crohn’s disease and ulcerative colitis among 25.1 million Medicare fee-for-service beneficiaries — United States, 2018 * Age-adjusted to the 2000 U.S. Census population aged ≥67 years based on three age groups (67–74, 75–84, and ≥85 years). https://data.census.gov/cedsci/table?t=Age%20and%20Sex&y=2000&d=DEC%20Summary%20File%201&tid=DECENNIALSF12000.PCT012&hidePreview=false ^†^ State-level age-adjusted prevalence estimate (%) was categorized into tertiles.

During 2001−2018, the overall prevalence of Crohn’s disease increased (APC = 3.4%, 95% CI = 3.2%–3.7%), as did the overall prevalence of ulcerative colitis (APC = 2.8%, 95% CI = 2.6%–3.0%) (Figure 2). The rate of increase was highest among non-Hispanic Black persons (APC = 5.0% for Crohn’s disease and 3.5% for ulcerative colitis) than it was among non-Hispanic White, Hispanic, and A/PI persons (APC range = 2.7%–3.5% for Crohn’s disease and 1.8%–2.9% for ulcerative colitis) (Figure 2). Prevalence estimates for both diseases were consistently highest among non-Hispanic White persons. The estimated prevalence of Crohn’s disease was consistently lowest among non-Hispanic A/PI persons. The estimated prevalence of ulcerative colitis was consistently higher among Hispanic persons than it was among members of other racial and ethnic minority groups.

**FIGURE 2:**
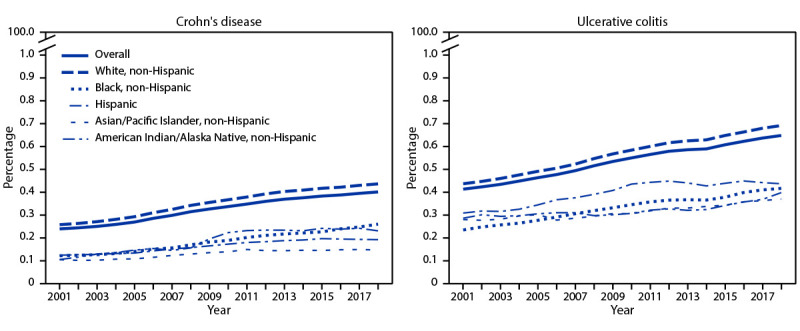
Age-adjusted prevalence*^,†^ of Crohn’s disease and ulcerative colitis among Medicare fee-for-service beneficiaries — United States, 2001—2018§ **Abbreviation:** APC = annual percentage change. * Age-adjusted to the 2000 U.S. Census population aged ≥67 years based on three age groups (67–74, 75–84, and ≥85 years). https://data.census.gov/cedsci/table?t=Age%20and%20Sex&y=2000&d=DEC%20Summary%20File%201&tid=DECENNIALSF12000.PCT012&hidePreview=false ^†^ Trends in age-adjusted prevalence estimates were assessed in linear regressions weighted with the estimates-associated inversed standard errors. The estimated prevalence was natural logarithm transformed. For Crohn’s disease, APC = 3.4% for overall, 3.5% for non-Hispanic White persons, 5.0% for non-Hispanic Black persons, 3.2% for Hispanic persons, 2.7% for Asian/Pacific Islander persons, and 5.3% for American Indian/Alaska Native persons. For ulcerative colitis, APC = 2.8% for overall, 2.9% for non-Hispanic White persons, 3.5% for non-Hispanic Black persons, 2.5% for Hispanic persons, 1.8% for Asian/Pacific Islander persons, and 1.5% for American Indian/Alaska Native persons. All were statistically significant (p<0.001). ^§ ^The conversion from the *International Classification of Diseases, Ninth Revision* diagnosis codes to the *International Classification of Diseases, Tenth Revision* diagnosis codes occurred on October 1, 2015.

## Discussion

During 2001−2018, the overall estimated prevalence of Crohn’s disease and ulcerative colitis among Medicare fee-for-service beneficiaries increased. These trends are consistent with those observed worldwide (*1*). Prevalence of both diseases was consistently highest among non-Hispanic White persons. However, the annual percentage increase in prevalence of ulcerative colitis was highest among non-Hispanic Black persons, and the increase in prevalence for Crohn’s disease was higher among non-Hispanic Black persons and among AI/AN persons than it was among non-Hispanic White persons. The potential rapid increase of disease prevalence in certain racial and ethnic minority groups indicates the need for tailored disease management strategies in these populations.

Racial/ethnic disparities have been noted in health care access, quality, and outcomes of patients with IBD (*3*). For example, hospitalization and mortality rates were higher among non-Hispanic Black patients than they were among non-Hispanic White patients. Non-Hispanic Black patients were more likely to have severe disease activity and were less likely to maintain medical therapy for IBD or to undergo surgery (*3*). Health literacy about IBD was also lower among non-Hispanic Black persons and Hispanic persons than it was among non-Hispanic White persons (*3*). These findings could help researchers understand racial/ethnic disparities in timing of diagnosis, health care access and use, and health literacy to promote health equity for IBD management in racial and ethnic minority groups.

Although the incidence of IBD peaks at approximately age 15–29 years (*4*), 10%–15% of new diagnoses occur among adults aged ≥60 years (*5*). Because overall mortality among patients with IBD is similar to that among the general U.S. population (*6*), prevalence is expected to increase as the U.S. population ages. In addition, the evolving therapeutic paradigm and more advanced diagnostic tools to detect the disease might also contribute to the increasing prevalence trends (*1*). The rise in prevalence could impose substantial financial costs on the health care system (*1*). Patients with IBD are at risk for impaired quality of life (*7*) because of the complexity of this lifelong disease, the potential adverse effects of treatment, and the fact that they tend to have more comorbidities than do patients without IBD (*2*), especially as they age.

The higher prevalence of IBD that was observed in women and in states in the Northeast region is consistent with a previous study (*8*). In addition, the current study found that the prevalence estimates of both diseases generally increased with a higher degree of urbanization. Living in urban areas, especially during early life, might be associated with risk for IBD through effects on the microbiome by factors such as pollution, diet, or lifestyle (*9*). The higher prevalence in large fringe metropolitan counties compared with large central metropolitan counties might be explained by the higher percentage of non-Hispanic White persons in large fringe metropolitan counties (*10*).

The findings in the report are subject to at least three limitations. First, Medicare data are collected for insurance reimbursement purposes. Therefore, certain socioeconomic measures, such as income and education, could not be assessed. Second, diagnosis codes related to Crohn’s disease or ulcerative colitis might be subject to coding errors. Finally, the study population was limited to Medicare fee-for-service beneficiaries (67% of all Medicare beneficiaries), and the findings might not be generalizable to all older adults in the United States.

Despite the limitations, Medicare data are a useful resource to monitor prevalence of IBD over time, understand its prevalence among older adults, assess differences by demographic and geographic characteristics, and have rich information to study health care use. Understanding temporal trends, especially the rate of increase among certain racial and ethnic minority groups, is important for resource planning and efforts to reduce health disparities. For optimal disease management, older adults of all races and ethnicities who have IBD should have routine doctor visits, adhere to a medication regimen, receive recommended preventive care, and adopt a healthy lifestyle, such as eating a well-balanced diet and quitting smoking for those who currently smoke.^¶^

**Summary**

**What is already known about this topic?**

Inflammatory bowel disease (IBD) prevalence is higher among non-Hispanic White persons than it is among persons in other racial/ethnic groups.

**What is added by this report?**

In 2018, 0.40% and 0.64% of 25.1 million Medicare fee-for-service beneficiaries aged ≥67 years had received a diagnosis of Crohn’s disease or ulcerative colitis. From 2001 to 2018, the age-adjusted prevalence of IBD increased among all racial/ethnic groups; the highest annual percentage increase was among non-Hispanic Black persons.

**What are the implications for public health practice?**

The study findings of increasing prevalence among older adults across all racial/ethnic groups, especially the higher rate of increase among certain racial and ethnic minority groups, underscore the importance for promoting health equity, guiding efforts to tailor disease management strategies for different populations, and continuing to monitor the temporal trends of the disease.

## Footnotes

* https://resdac.org/cms-data/files/medpar; https://resdac.org/cms-data/files/carrier-ffs; https://www.resdac.org/cms-data/files/op-ffs

†
https://www.cdc.gov/nchs/data/series/sr_02/sr02_166.pdf

§ Direct age adjustment according to 2000 U.S. Census population (https://data.census.gov/cedsci/table?t=Age%20and%20Sex&y=2000&d=DEC%20Summary%20File%201&tid=DECENNIALSF12000.PCT012&hidePreview=false) based on three age groups (67−74, 75−84, and ≥85 years).

¶
https://www.cdc.gov/dotw/ibd/index.html

## References

[1] Alatab S, Sepanlou SG, Ikuta K, ; GBD 2017 Inflammatory Bowel Disease Collaborators. The global, regional, and national burden of inflammatory bowel disease in 195 countries and territories, 1990–2017: a systematic analysis for the Global Burden of Disease Study 2017. Lancet Gastroenterol Hepatol 2020;5:17–30. 10.1016/S2468-1253(19)30333-4 31648971 PMC7026709

[2] Xu F, Dahlhamer JM, Zammitti EP, Wheaton AG, Croft JB. Health-risk behaviors and chronic conditions among adults with inflammatory bowel disease—United States, 2015 and 2016. MMWR Morb Mortal Wkly Rep 2018;67:190–5. 10.15585/mmwr.mm6706a4 29447146 PMC5815485

[3] Sewell JL, Velayos FS. Systematic review: the role of race and socioeconomic factors on IBD healthcare delivery and effectiveness. Inflamm Bowel Dis 2013;19:627–43. 10.1002/ibd.22986 22623078 PMC3905682

[4] Johnston RD, Logan RF. What is the peak age for onset of IBD? Inflamm Bowel Dis 2008;14(Suppl 2):S4–5. 10.1002/ibd.20545 18816745

[5] Taleban S, Colombel JF, Mohler MJ, Fain MJ. Inflammatory bowel disease and the elderly: a review. J Crohn’s Colitis 2015;9:507–15. 10.1093/ecco-jcc/jjv059 25870198

[6] Aniwan S, Harmsen WS, Tremaine WJ, Kane SV, Loftus EV Jr. Overall and cause-specific mortality of inflammatory bowel disease in Olmsted county, Minnesota, from 1970 through 2016. Mayo Clin Proc 2018;93:1415–22. 10.1016/j.mayocp.2018.03.004 30293558 PMC6178953

[7] Knowles SR, Graff LA, Wilding H, Hewitt C, Keefer L, Mikocka-Walus A. Quality of life in inflammatory bowel disease: a systematic review and meta-analyses. Part I. Inflamm Bowel Dis 2018;24:742–51. 10.1093/ibd/izx100 29562277

[8] Kappelman MD, Moore KR, Allen JK, Cook SF. Recent trends in the prevalence of Crohn’s disease and ulcerative colitis in a commercially insured US population. Dig Dis Sci 2013;58:519–25. 10.1007/s10620-012-2371-5 22926499 PMC3576554

[9] Benchimol EI, Kaplan GG, Otley AR, Rural and urban residence during early life is associated with risk of inflammatory bowel disease: a population-based inception and birth cohort study. Am J Gastroenterol 2017;112:1412–22. 10.1038/ajg.2017.208 28741616 PMC5596205

[10] Ingram DD, Franco SJ. 2013 NCHS urban-rural classification scheme for counties. Vital Health Stat 2014; 2(166):1–73. 24776070

